# Refining minimal engineered receptors for specific activation of on-target signaling molecules

**DOI:** 10.1038/s41598-024-81259-4

**Published:** 2024-12-30

**Authors:** Masashi Miura, Masahiro Kawahara

**Affiliations:** Laboratory of Cell Vaccine, Microbial Research Center for Health and Medicine (MRCHM), National Institutes of Biomedical Innovation, Health and Nutrition (NIBIOHN), 7-6-8 Saito-Asagi, Ibaraki-Shi, Osaka, 567-0085 Japan

**Keywords:** Biotechnology, Molecular engineering, Protein design, Synthetic biology, Genetic engineering

## Abstract

Since designer cells are attracting much attention as a new modality in gene and cell therapy, it would be advantageous to develop synthetic receptors that recognize artificial ligands and activate solely signaling molecules of interest. In this study, we refined the construction of our previously developed minimal engineered receptors (MERs) to avoid off-target activation of STAT5 while maintaining on-target activation of signaling molecules corresponding to tyrosine motifs. Among the myristoylated, cytoplasmic, and transmembrane types of MERs, the cytoplasmic type had the highest signaling efficiency, although there was off-target activation of STAT5 upon ligand stimulation. Tyrosine-to-phenylalanine ​​mutagenesis revealed that both the tyrosine motif for recruiting target signaling molecules and the tyrosine residues in the JAK-binding domain did not contribute to off-target activation of STAT5. Using alanine mutagenesis for Box1 of the JAK-binding domain of MERs, we ultimately found a Box1 mutation that slightly reduced activation of on-target signaling molecules but minimized off-target activation of STAT5. The refined MER enabled us to precisely analyze the signaling and cell fate-inducing properties of seven tyrosine motifs. Therefore, the refined MER, which realizes activation of on-target signaling molecules with high signal-to-noise ratios, will attract much attention as a tool for functionalizing designer cells and more broadly in the field of synthetic biology.

## Introduction

Cells receive signals from the extracellular milieu and initiate intracellular signaling to determine their fate. This process is crucial for cells to play various important roles as members of our body. For example, under hypoxic conditions, erythropoietin is produced from the kidney, which acts on erythropoietin receptors expressed on myeloid progenitors in the bone marrow to promote erythroblast differentiation through intracellular signaling^1–3^. Upon microbial or viral infections, antigen-presenting cells display peptides on major histocompatibility complex class II (MHC-II), whereas helper T cells recognize the peptide-MHC-II complexes through specific T cell receptors, secrete cytokines through intracellular signaling, and activate antigen-specific B cells, thereby inducing adaptive immunity^4–6^. Thus, various receptors function as a conductor for orchestrating intracellular signaling to induce desired cell fate.

Recently, designer cells are attracting much attention as a new modality in gene and cell therapy^7–9^. In order to precisely control fates of designer cells without affecting normal cells, it would be advantageous to develop synthetic receptors that recognize artificial ligands and activate solely signaling molecules of interest^10–13^. To this end, we previously created synthetic receptors, hereafter designated as minimal engineered receptors (MERs), by rationally arranging molecular parts in a bottom-up manner^14^. The signaling domain of MERs consists of a Janus tyrosine kinase (JAK)-binding domain, a tyrosine motif that recruits signaling molecules with Src homology 2 (SH2) or phosphotyrosine binding (PTB) domains only when tyrosine-phosphorylated, and a minimal linker sequence that connects multiple tyrosine motifs. In order to activate JAK, the signaling domain was fused with myristoylation and dimerization domains for membrane localization and synthetic ligand-dependent activation, respectively. When MERs were expressed by genetic modification of cells and stimulated with a synthetic ligand, MERs successfully activated desired signaling molecules corresponding to the tyrosine motifs connected^14^.

However, there was a problem of MERs to be solved. The activation of MERs was always accompanied by off-target activation of STAT5; *i.e.* STAT5 was activated even when the STAT5-binding motif was absent from MERs. This phenomenon hampers a programmable nature of MERs, in which the signaling properties of MERs could be readily modulated by choosing appropriate tyrosine motifs. It would be desirable to minimize the off-target activation of STAT5 especially for such experiments that a specific signaling molecule is activated by MER to determine what kind of cellular phenotype could be induced. Therefore, in this study we aim to refine construction of MERs to minimize off-target activation of STAT5. We also incorporate arbitrary tyrosine motifs to the refined MER and analyze their signaling and cell fate-inducing properties as precisely as possible.

## Results

### Differential localization of MERs revealed significantly different signaling intensities

**Fig. 1:**
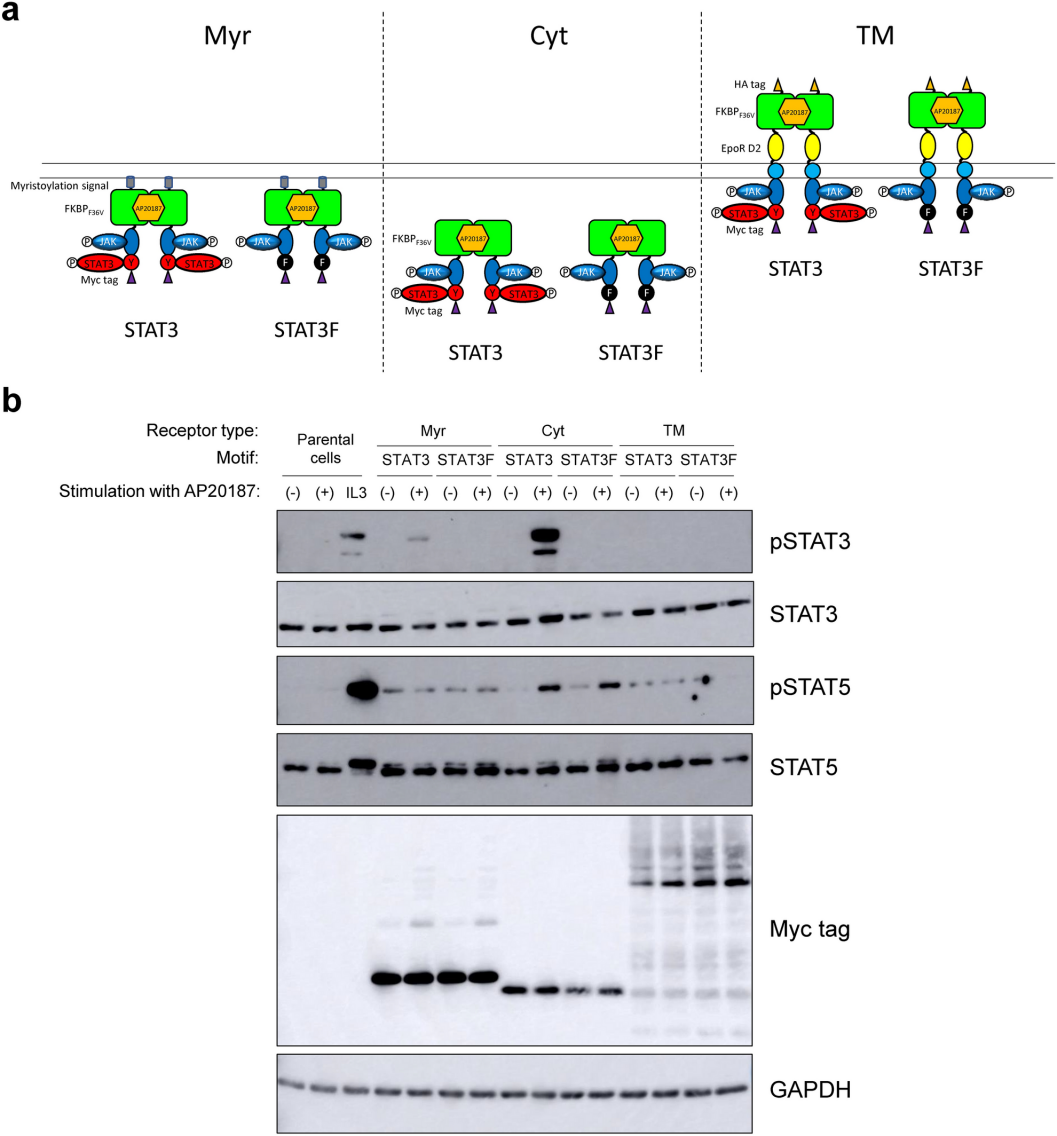
Differential localization of MERs revealed significantly different signaling intensities. (**a**) The molecular architecture of the constructed MERs. The myristoylated (Myr), cytoplasmic (Cyt), and transmembrane (TM) type receptors were designed. The STAT3-binding motif (STAT3) or its tyrosine-to-phenylalanine mutant (STAT3F) were incorporated into the signaling domain of the MERs. (**b**) Signaling analysis. Parental Ba/F3 cells and the transductants were stimulated with no ligand (-), 50 nM AP20187 (+), or 1 ng/mL IL-3 (IL3). Blots with the phospho-specific antibodies detected activation levels of signaling molecules, whereas blots with anti-Myc tag detected expression levels of the MERs (n = 1). Blots with anti-whole signaling molecules and anti-GAPDH serve as loading controls (n = 1). Uncropped blot images are provided in Supplementary Information (Supplementary Fig. 6).

The MERs constructed in our previous study consisted of a myristoylation signal sequence, a dimerization domain FKBP_F36V_, the JAK-binding domain of c-mpl, and tyrosine motifs, hereafter designated as a Myr type^14^. The Myr-type MERs showed off-target activation of STAT5 even in the absence of AP20187, which is a synthetic dimerizer ligand for FKBP_F36V_. This off-target activation of STAT5 may be due to either direct activation of STAT5 by JAK^15,16^ or nonspecific binding of STAT5 to the tyrosine motifs within the MERs. In this study, we constructed a series of MERs to examine these possibilities (Fig. 1a). First, we incorporated a STAT3-binding motif or its tyrosine-to-phenylalanine mutant as a tyrosine motif of MERs, aiming to compare the signaling properties in parallel. In addition, since off-target activation of STAT5 in the absence of ligand stimulation may be caused by increased frequency of collisions of MERs through membrane localization, we also constructed a non-membrane-localized cytoplasmic (Cyt) type and in contrast a transmembrane (TM) type. As for the extracellular domain of the TM type, the EpoR D2 domain in addition to FKBP_F36V_ was incorporated because our previous studies showed that receptor constructs using the EpoR D2 domain resulted in sufficient expression and signaling of various antibody-receptor chimeras^17–27^.

IL-3-dependent Ba/F3 cells were transduced with retroviral vectors encoding these MERs, and stable transductants were obtained by antibiotic selection. The cells were depleted in IL-3-free medium and stimulated with AP20187 to activate the MERs (Fig. 1b). Western blotting confirmed expression of all three types of the MERs at the expected molecular sizes. Signaling analysis showed that the Myr and Cyt types activated the target signaling molecule STAT3, with the Cyt type being particularly efficient, whereas the TM type was unable to activate STAT3. The off-target activation of STAT5 was ligand-independent in the Myr and TM types. The Cyt type had the strongest off-target activation of STAT5 with ligand stimulation, but the weakest off-target activation of STAT5 without ligand stimulation. In addition, even when the STAT3-binding motif was functionally knocked out by the tyrosine-to-phenylalanine mutation, off-target activation of STAT5 still occurred, indicating that off-target activation of STAT5 is not due to the tyrosine motif. These results suggest that off-target activation of STAT5 occurs due to enhanced receptor–receptor association through membrane localization in the Myr and TM types, whereas off-target activation of STAT5 occurs in the Cyt type when JAK is activated in a ligand-dependent manner.

### Two tyrosines in the JAK-binding domain do not contribute to off-target activation of STAT5

**Fig. 2:**
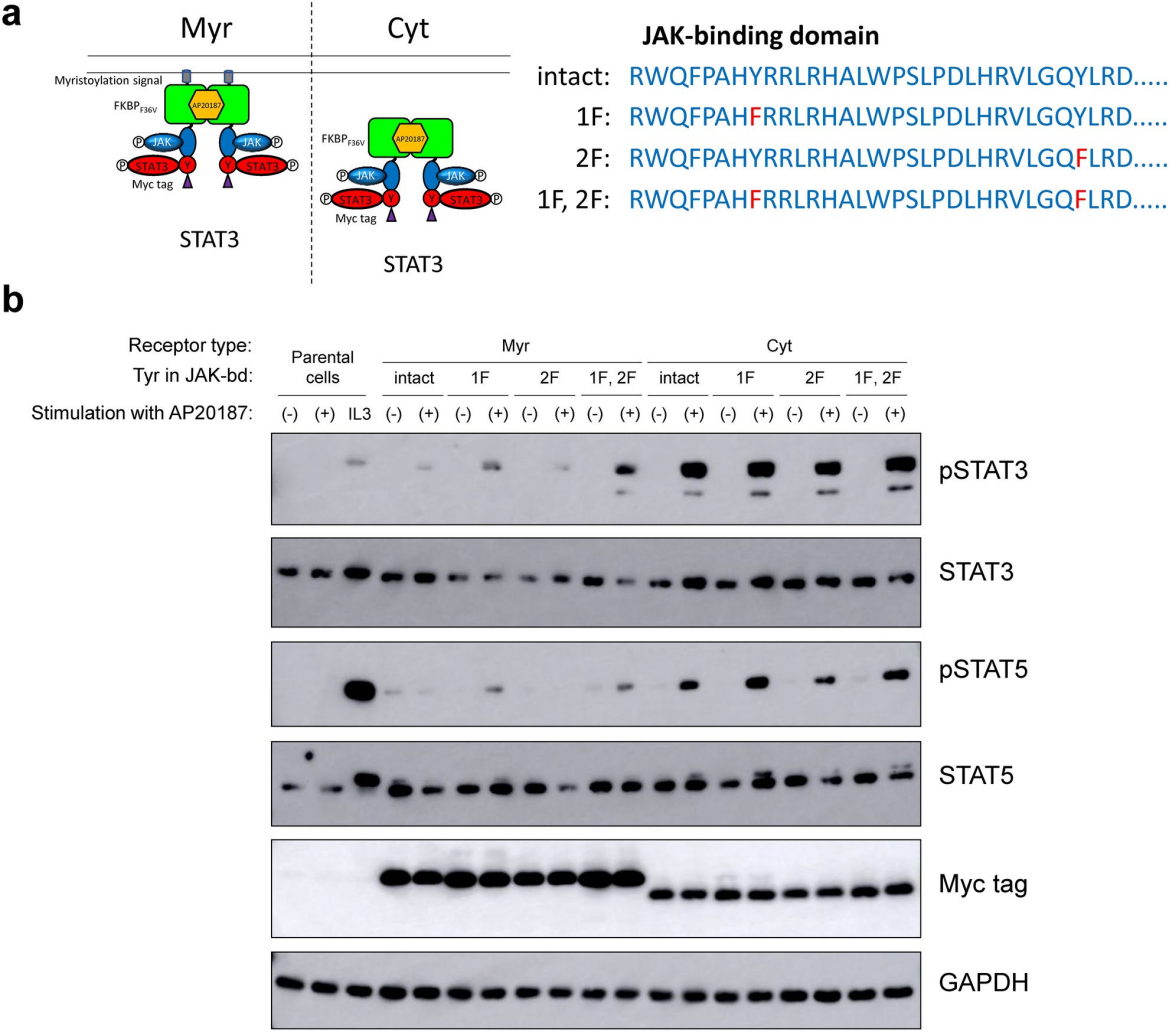
Two tyrosines in the JAK-binding domain do not contribute to off-target activation of STAT5. (**a**) The molecular architecture of the constructed MERs. One or both of the two tyrosine residues in the JAK-binding domain were mutated to phenylalanine in the Myr- and Cyt-type MERs. The STAT3-binding motif (STAT3) was incorporated into the signaling domain of the MERs. (**b**) Signaling analysis. Parental Ba/F3 cells and the transductants were stimulated with no ligand (-), 50 nM AP20187 (+), or 1 ng/mL IL-3 (IL3). Blots with the phospho-specific antibodies detected activation levels of signaling molecules, whereas blots with anti-Myc tag detected expression levels of the MERs (n = 1). Blots with anti-whole signaling molecules and anti-GAPDH serve as loading controls (n = 1). Uncropped blot images are provided in Supplementary Information (Supplementary Fig. 7).

Although the experiments in the previous section revealed that off-target activation of STAT5 was not due to the tyrosine motif, another possibility is that the two tyrosine residues in the JAK-binding domain may function as cryptic tyrosine motifs and recruit STAT5 upon phosphorylation. To test this possibility, one or both of the two tyrosine residues were mutated to phenylalanine in the Myr- and Cyt-type MERs (Fig. 2a). Signaling analysis showed that even when both tyrosine residues were mutated to phenylalanine, on-target activation of STAT3 and off-target activation of STAT5 occurred at almost the same levels as those observed in the intact constructs (Fig. 2b). Therefore, the results proved that the two tyrosine residues neither affect the activation levels of JAK nor involve off-target activation of STAT5. However, since the two tyrosine residues might cause off-target activation of signaling molecules other than STAT5, hereafter we utilized the mutated JAK-binding domain in which both tyrosine residues were mutated to phenylalanine. With regard to the receptor type, we chose the Cyt type, which attained high phosphorylation levels of the on-target signaling molecule.

### Box1 point mutations modulate on-target/off-target ratio

**Fig. 3:**
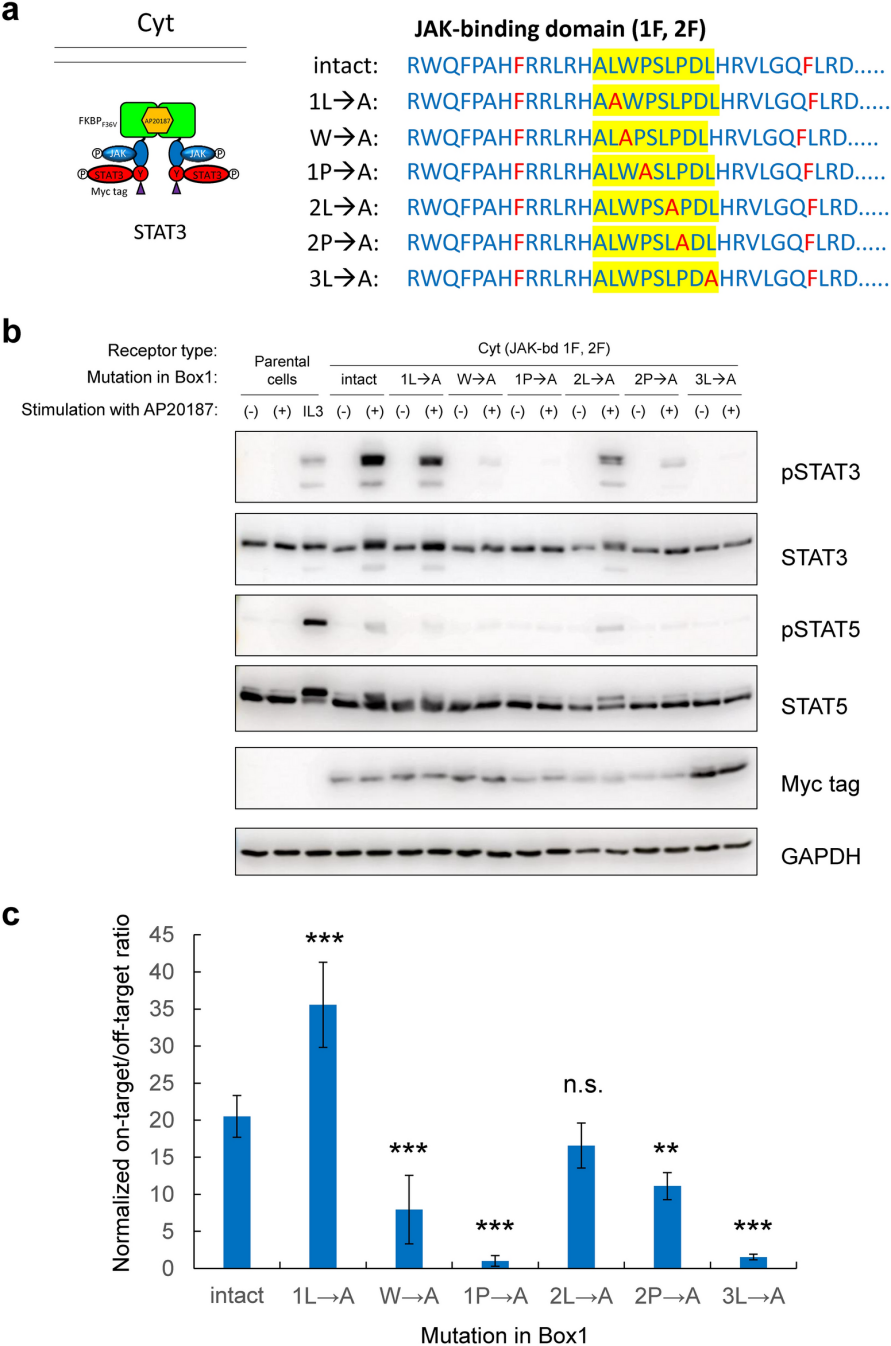
Box1 point mutations modulate on-target/off-target ratio. (**a**) The molecular architecture of the constructed MERs. Six highly conserved consensus amino acid residues in Box1 (filled in yellow) were point-mutated to alanine in the Cyt-type MER with two tyrosine-to-phenylalanine mutations of the JAK-binding domain. The STAT3-binding motif (STAT3) was incorporated into the signaling domain of the MERs. (**b**) Signaling analysis. Parental Ba/F3 cells and the transductants were stimulated with no ligand (-), 50 nM AP20187 (+), or 1 ng/mL IL-3 (IL3). Blots with the phospho-specific antibodies detected activation levels of signaling molecules, whereas blots with anti-Myc tag detected expression levels of the MERs (n = 1). Blots with anti-whole signaling molecules and anti-GAPDH serve as loading controls (n = 1). Uncropped blot images are provided in Supplementary Information (Supplementary Fig. 8). (**c**) Quantification of the on-target/off-target ratios from the blotting data. The uncropped blot images of additional 4 gel blots for pSTAT3/STAT3 and pSTAT5/STAT5 are provided in Supplementary Information (Supplementary Fig. 9). The phosphorylation level of each signaling molecule with AP20187 stimulation was normalized by the value of IL-3-stimulated parental cells, and further normalized by the expression level in order to enable comparison among the multiple gel blots. The on-target/off-target ratios were calculated by dividing the normalized pSTAT3 values by the normalized pSTAT5 values. The data are represented as mean ± SD (n = 5). One-way ANOVA followed by post-hoc Bonferroni’s multiple-comparison test was used for comparing the means of the groups. ***p < 0.001; **p < 0.01; n.s., not significant versus intact.

In the experiments described in the previous section, off-target activation of STAT5 was observed even when all tyrosine residues in the MERs were removed, indicating that it was due to direct activation of STAT5 by JAK. Therefore, it is necessary to lower the off-target activation level of STAT5 while maintaining on-target activation levels by modulating activation modes of JAK. The amino acid residues in Box1 of the JAK-binding domain are known to be critical for JAK binding^16,28–30^. Therefore, we aimed to screen for Box1 point mutants with high on-target/off-target ratios. Six highly conserved consensus amino acid residues in Box1^28^ were point-mutated to alanine (Fig. 3a), and transductants expressing each of the mutants were subjected to signaling analysis. As a result, when W, 1P, 2P, and 3L were mutated to alanine, on-target activation of STAT3 was significantly reduced, while off-target activation of STAT5 was eliminated (Fig. 3b). On the other hand, when 1L was mutated to alanine, on-target activation of STAT3 was almost maintained, and off-target activation of STAT5 was almost abolished. When 2L was mutated to alanine, both on-target activation of STAT3 and off-target activation of STAT5 were reduced. To confirm these observations quantitatively, we performed experiments to obtain additional 4 gel blots for pSTAT3/STAT3 and pSTAT5/STAT5 and quantified the bands (Fig. 3c). As a result, the on-target/off-target ratios changed depending on the Box1 mutants, among which the 1L→A mutant gave the highest value. Furthermore, the difference between the intact and 1L→A mutant was statistically significant. Thus, the 1L→A mutant revealed desirable signaling properties with a better on target/off target ratio than the intact.

### The refined MER enables analysis of signaling properties of arbitrary tyrosine motifs

We aimed to incorporate a series of tyrosine motifs (with putative specificity toward STAT1 to 6 and Shc) into the refined Cyt-type MER variant (JAK-bd 1F, 2F / Box1 1L→A) and evaluate the signaling properties of each tyrosine motif. The STAT1-, STAT3-, STAT5-, and Shc-binding motifs, which can preferentially activate the corresponding signaling molecules, are the same as those used in our previous study^10,14^. On the other hand, the STAT2-, STAT4-, and STAT6-binding motifs are chosen based on literature and used for the first time in this study. The STAT2-binding motif is derived from IFNAR2 and has been shown to activate not only STAT2 but also STAT1 and STAT3^31^. The STAT4- and STAT6-binding motifs are derived from IL-12Rβ2 and IL-4Rα, respectively, but specificities of the motifs are unknown^32,33^.

**Fig. 4:**
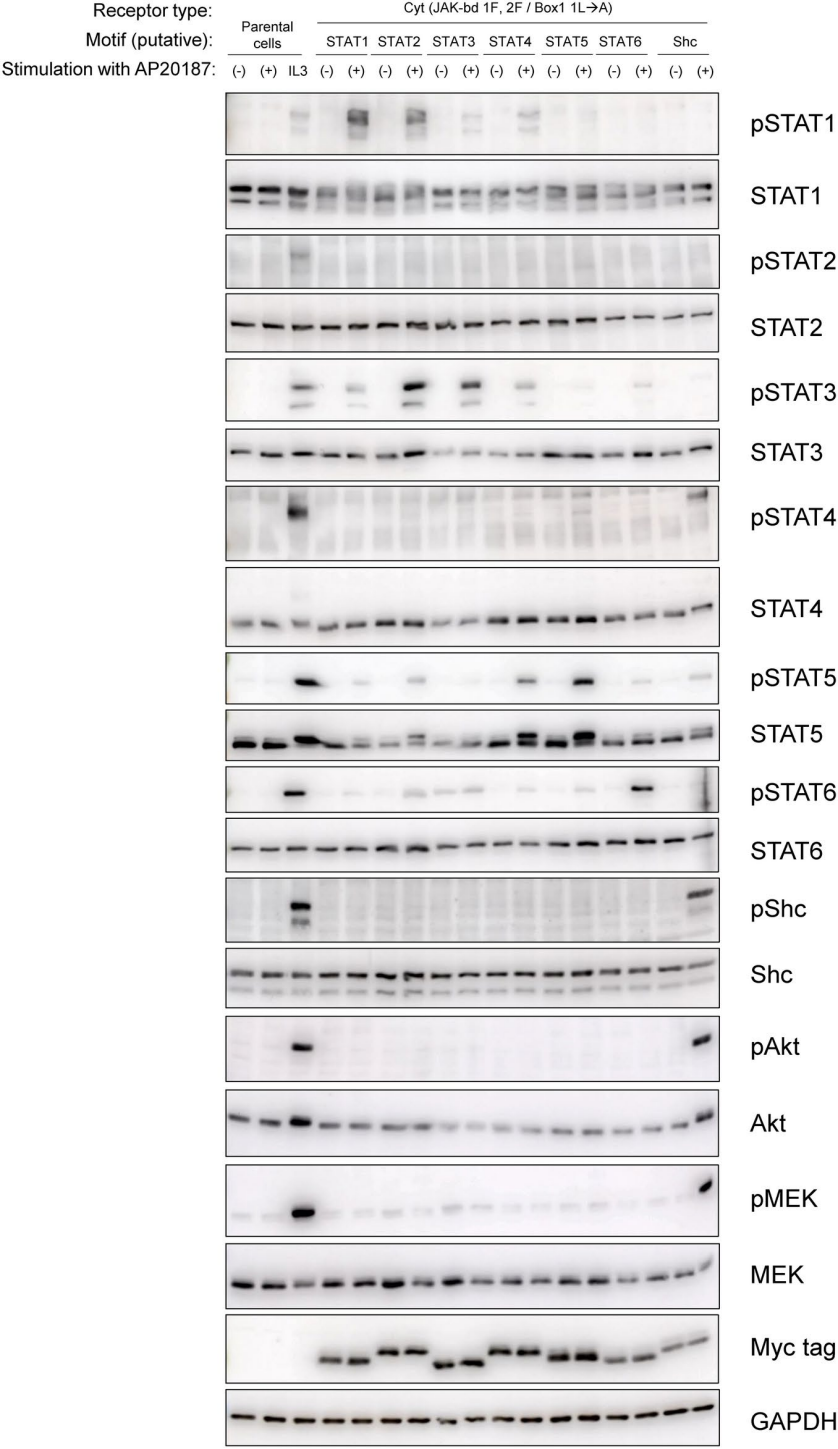
The refined MER enables analysis of signaling properties of arbitrary tyrosine motifs. Parental Ba/F3 cells and the transductants with putative STATs- and Shc-binding motifs in the refined MER were stimulated with no ligand (-), 50 nM AP20187 ( +), or 1 ng/mL IL-3 (IL3). Blots with the phospho-specific antibodies detected activation levels of signaling molecules, whereas blots with anti-Myc tag detected expression levels of the MERs (n = 1). Blots with anti-whole signaling molecules and anti-GAPDH serve as loading controls (n = 1). Uncropped blot images and a graph qualitatively illustrating the results are provided in Supplementary Information (Supplementary Figs. 10 and 11).

We established stable transductants expressing the MERs incorporating these motifs and examined their signaling properties by Western blotting. Consequently, corresponding bands were observed correctly for the phospho-STAT1, 3, 5, 6, and Shc, whereas no corresponding bands were observed at the expected molecular sizes for the phospho-STAT2 and 4 (Fig. 4). The specificities of the putative STAT2- and STAT4-binding motifs were insufficient, activating all of STAT1, 3, 5, and 6. In contrast, the putative STAT1-, 3-, 5-, 6-, and Shc-binding motifs activated the corresponding signaling molecules more specifically, although non-specific activation of the other signaling molecules was observed at weak levels. Only the Shc-binding motif activated Akt and MEK, indicating that the STATs-binding motifs did not recruit adapter molecules that activate the Akt pathway and/or the Ras/MAPK pathway. Thus, the STATs-binding motifs revealed to be specific to STATs, but could be of a promiscuous nature within STATs depending on the motifs.

### Phenotypic assays revealed differential cell fate-inducing properties among the motifs

**Fig. 5:**
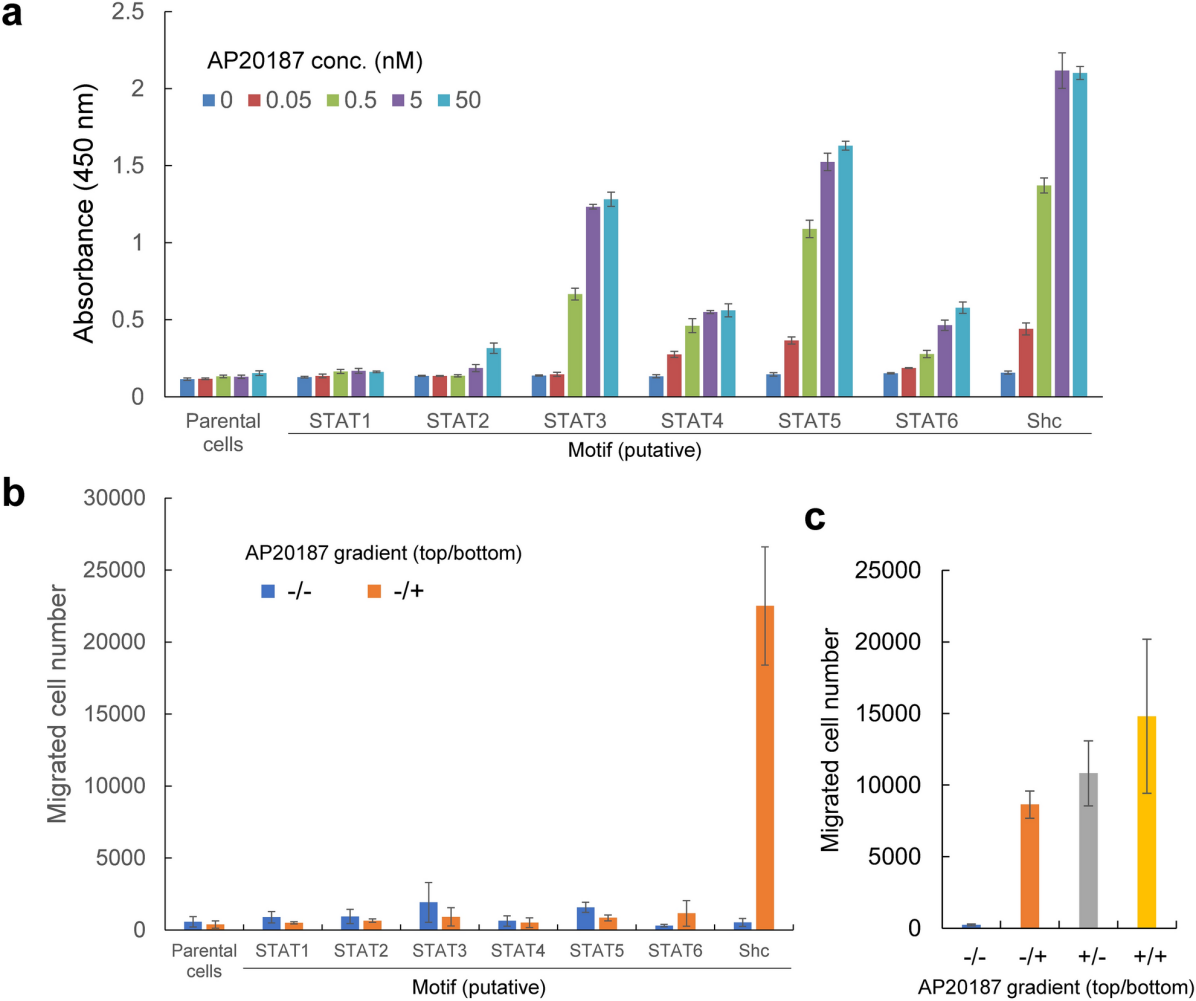
Phenotypic assays revealed differential cell fate-inducing properties among the motifs. (**a**) Cell proliferation assay. Cells were cultured with the indicated concentrations of AP20187 for 3 days, and cell proliferation levels were measured by a colorimetric assay. The data are represented as mean ± SD (n = 3, triplicate cultures). (**b**, **c**) Cell migration assay. Cells (3 × 10^5^) were added to the top chambers of 24-well transwell plates. Cell migration to the bottom chambers after incubation for 5 h was measured by counting with a flow cytometer. The data represent mean ± SD (n = 3, triplicate wells). (**b**) The ligand AP20187 (50 nM) was added to the bottom chambers to create positive gradients. (**c**) The cells expressing the MER with the Shc-binding motif were subjected to this assay. AP20187 (50 nM) was added to the bottom and/or top chambers to create positive (top/bottom: − / +), negative (+ / −), and no (− / − and + / +) gradients.

To analyze cell fate-inducing properties of the MERs incorporating the tyrosine motifs, we performed phenotypic assays on proliferation, migration, and motility. A cell proliferation assay showed that ligand-dependent cell proliferation was strongly induced by the STAT3-, STAT5-, and Shc-binding motifs, weakly induced by the STAT4- and STAT6-binding motifs, and barely induced by the STAT1- and STAT2-binding motifs (Fig. 5a). A cell migration assay using transwell plates showed that cell migration was induced in response to a positive gradient of the ligand only by the Shc-binding motif (Fig. 5b). To verify whether the cell migration induced by the Shc-binding motif was chemotactic or chemokinetic, four gradient patterns were created in the top and bottom chambers with and without the ligand. Consequently, cell migration occurred even when the ligand was added to the top chamber, demonstrating a chemokinetic nature of the cell migration (Fig. 5c). Time-lapse observation of cell motility in a horizontal chamber showed that cell motility was significantly enhanced in response to the ligand only by the Shc-binding motif (Supplementary Movies 1 to 16). In the signaling analysis shown in Fig. 4, the Akt and Ras/MAPK pathways were activated only by the Shc-binding motif, which coincides the results of cell migration and motility assays. Taken together, activation of the Ras/MAPK and Akt pathways was important for cell migration and motility, while the JAK/STAT pathway was not involved in these cellular phenotypes. These results demonstrate that the refined MER is useful for analyzing the signaling properties of tyrosine motifs and precisely inducing desired cellular phenotypes.

## Discussion

In this study, we refined the construction of MERs in order to avoid off-target activation of STAT5 while maintaining on-target activation of signaling molecules corresponding to tyrosine motifs. First, in parallel comparison of the Myr, Cyt, and TM types, we found that the Cyt type had the highest signaling efficiency, although there was off-target activation of STAT5 upon ligand stimulation. In addition, Y-to-F mutagenesis revealed that both the tyrosine motif for recruiting target signaling molecules and the tyrosine residues in the JAK-binding domain did not contribute to off-target activation of STAT5. Since Box1 is known to be important for JAK binding, we replaced the amino acid residues in the consensus sequence of Box1 with alanine, and ultimately found a Box1 mutation (the first leucine of Box1 was mutated to alanine) that slightly reduced activation of on-target signaling molecules but minimized off-target activation of STAT5. Presumably, this mutation changed the binding state and conformation of JAK, which in turn reduced off-target activation of STAT5.

The refined MER enabled us to analyze the signaling properties of seven tyrosine motifs. The STAT1-, 3-, 5-, 6-, and Shc-binding motifs activated corresponding signaling molecules almost specifically, whereas the STAT2- and STAT4-binding motifs promiscuously activated STATs. Shc is known to bind to the NPXY consensus motif through the PTB domain^34^, and our experimental results indicate that Shc does not bind to any of the six STAT-binding motifs. This suggests that Shc has strict specificity toward the NPXY consensus motif and thus cannot bind to motifs different from the consensus sequence. Conversely, the Shc-binding motif did not activate any of the STATs, suggesting that the NPXY motif also has strict specificity toward Shc. On the other hand, STATs bind to the STAT-binding motifs through the SH2 domain. Our experimental results showing a promiscuous nature for activation of STATs indicate that STATs have moderate specificity toward the consensus sequences (*e.g.* YxxQ for STAT3; YxxL for STAT5) and vice versa. The extent of specificity between tyrosine motifs and signaling molecules could characterize signal transduction via the PTB and SH2 domains, and their differences and orthogonality may have physiological significance in cytokine receptor-mediated signal transduction. To demonstrate this speculation, ultimately the refined MERs should be tested with phosphoproteomics analysis to define the real extent of cross-specificity of investigated motifs.

It has been reported that activation of STAT3, STAT5, and Shc promotes proliferation of Ba/F3 cells, whereas activation of STAT1 rather inhibits the proliferation^35,36^. Consistent with this, the phenotypic assays showed that proliferation was observed for the motifs activating STAT3, STAT5, STAT6, or Shc (*i.e.* the putative STAT3-, 4-, 5-, 6-, and Shc-binding motifs), while proliferation was hardly observed for the motifs strongly activating STAT1 (*i.e.* the putative STAT1- and STAT2-binding motifs). Intriguingly, since the STAT6-binding motif hardly activated STAT3, STAT5, and Shc, the results proved that STAT6 promotes proliferation of Ba/F3 cells, which is a novel finding. Cell migration and motility assays clearly indicate that activation of the Ras/MAPK and Akt pathways following Shc activation is important for reorganizing the actin skeleton to enhance cell motility. Of note, the cell motility was not enhanced at all by activation of the JAK-STAT pathway. Thus, cell proliferation and motility properties could be separately induced by incorporating appropriate tyrosine motifs in the refined MER. This study may provide important clues for rationally designing synthetic receptors on the basis of cellular phenotypes of interest. Furthermore, engineered receptors may be an excellent tool to study protein motifs and signaling pathways in separation.

While we characterized the signaling and cell fate-inducing properties of seven tyrosine motifs using the refined MER, the assays could be extended to characterize various other tyrosine motifs. Furthermore, the target cells are not limited to Ba/F3 cells; the refined MER could be applied to various other cell types that are suitable for therapeutic applications. Therefore, the refined MER, which realizes activation of on-target signaling molecules with high signal-to-noise ratios, will attract much attention as a tool for functionalizing designer cells and more broadly in the field of synthetic biology.

## Materials and methods

### Plasmid construction

The MER genes were encoded in a retroviral plasmid pMK-stuffer-IRES-Puro^R^-T2A-EGFP^14^. Construction and subcloning of the plasmids encoding the MER genes were conducted as previously described^14^. Comprehensive representations for the amino acid sequences of MERs are provided in Supplementary Information (Supplementary Figs. 1 to 4).

### Gene transduction

An IL-3-dependent Ba/F3 cell line (#RCB0805; purchased from RIKEN Cell Bank, Ibaraki, Japan) was retrovirally transduced in a standard protocol as described previously^23^. Retroviral packaging cells seeded in 6-well plates were transiently transfected with 3 μg of the constructed plasmid, 7.5 μl of Lipofectamine LTX (Thermo Fisher Scientific, Waltham, MA), and 3 μl of Plus Reagent (Thermo Fisher Scientific). Two days later, the retroviral supernatant was adsorbed on 24-well plates precoated with RetroNectin (Takara Bio, Shiga, Japan) for 6 h. After removal of the retroviral supernatant from each well, Ba/F3 cells (1 × 10^5^ cells) were seeded for gene transduction. One day later, the transductants were selected by adding 2 µg/mL puromycin (Sigma-Aldrich, St. Louis, MO) in the culture medium.

### Signaling analysis

Cells were washed twice with PBS and cultured in a depletion medium (the culture medium without IL-3) for 6 h to deplete the signaling effects with IL-3. The cells were stimulated with no ligand, 50 nM AP20187 (B/B Homodimerizer; Takara Bio) or 1 ng/mL IL-3 at 37℃ for 15 min and immediately washed twice with 2 mM Na_3_VO_4_ in ice-cold PBS. Preparation of cell lysate and subsequent SDS-PAGE / Western blotting were conducted as described previously^24^. The cells were lysed in Cell Lysis Buffer M (Fujifilm Wako Pure Chemical, Osaka, Japan) with Protease Inhibitor Cocktail (Nacalai Tesque, Kyoto, Japan) and 1 mM Na_3_VO_4_. After incubating on ice for 10 min, the lysate was centrifuged at 22,300 g for 10 min at 4℃. The supernatant was mixed with 4X SDS-PAGE Sample Buffer (Tokyo Chemical Industry, Tokyo, Japan) and boiled at 98℃ for 5 min. Proteins of the cell lysates were resolved by SDS-PAGE and transferred on a nitrocellulose membrane. After the membrane was blocked with 3% BSA (Nacalai Tesque), the blots were probed with rabbit primary antibodies and HRP-conjugated anti-rabbit secondary antibody. Chemiluminescence was generated by Immobilon Forte Western HRP substrate (Merck-Millipore, Burlington, MA) and detected by ImageQuant LAS 500 (Cytiva, Marlborough, MA). The band intensities were quantified using ImageQuantTL software (Cytiva). The antibodies used are listed in Supplementary Information (Supplementary Fig. 5).

### Cell proliferation assay

Cells were washed twice with PBS and seeded into 24-well plates with various concentrations of AP20187 at the initial cell density of 3 × 10^4^ cells/mL. On day 3, viable cell densities were estimated with the use of Cell Counting Kit-8 (Dojindo Laboratories, Kumamoto, Japan), whose readout (absorbance at 450 nm) was measured by GloMax Discover Microplate Reader (Promega, Madison, WI).

### Cell migration assay

Cells were washed twice with PBS and cultured in the depletion medium for 16 h. For no-gradient samples, cells (3 × 10^5^) were resuspended in 200 µL of the depletion medium and added to the top chamber of 24-well transwell plates with 5 µm of a membrane pore (Kurabo, Osaka, Japan), whereas 600 µL of the depletion medium was added to the bottom chamber. AP20187 was added to the bottom/top chambers to create positive/negative gradients. After incubation for 5 h, the number of cells migrated into the bottom chamber was measured by counting with a flow cytometer FACSCalibur (Becton Dickinson, Lexington, KY).

### Cell motility assay

Cells were washed twice with PBS and seeded into 24-well plates at 5 × 10^5^ cells/well in 500 μL of the depletion medium for 16 h. The cells were mixed with 500 μL of the depletion medium to become final concentration of 0 or 50 nM AP20187. Ten microliters of the cell samples were pipetted into Countess Cell Counting Chamber Slides (Thermo Fisher Scientific), and time-lapse images of the cells were acquired at the intervals of 30 s for 1 h using CytoWatcher (ATTO, Tokyo, Japan) in the incubator. Movies were generated by connecting the time-lapse images at 20 frames/sec using ImageJ software.

## Supplementary Information

Supplementary Information 1.

Supplementary Information 2.

## Data Availability

All data generated or analyzed during this study are included in this published article and its Supplementary Information.

## References

[1] Haase, V. H. Regulation of erythropoiesis by hypoxia-inducible factors. *Blood Rev.* **27**, 41–53 (2013). 23291219 10.1016/j.blre.2012.12.003 PMC3731139

[2] Tsiftsoglou, A. S. Erythropoietin (EPO) as a key regulator of erythropoiesis, bone remodeling and endothelial transdifferentiation of multipotent mesenchymal stem cells (MSCs): Implications in regenerative medicine. *Cells* **10**, 2140 (2021). 34440909 10.3390/cells10082140 PMC8391952

[3] Watts, D. et al. Hypoxia pathway proteins are master regulators of erythropoiesis. *Int. J. Mol. Sci.* **21**, 8131 (2020). 33143240 10.3390/ijms21218131 PMC7662373

[4] Bretou, M. et al. Dynamics of the membrane-cytoskeleton interface in MHC class II-restricted antigen presentation. *Immunol. Rev.* **272**, 39–51 (2016). 27319341 10.1111/imr.12429

[5] Katikaneni, D. S. & Jin, L. B cell MHC class II signaling: A story of life and death. *Hum. Immunol.* **80**, 37–43 (2019). 29715484 10.1016/j.humimm.2018.04.013 PMC6207480

[6] Unanue, E. R., Turk, V. & Neefjes, J. Variations in MHC class II antigen processing and presentation in health and disease. *Annu. Rev. Immunol.* **34**, 265–297 (2016). 26907214 10.1146/annurev-immunol-041015-055420

[7] Kimbrel, E. A. & Lanza, R. Next-generation stem cells - ushering in a new era of cell-based therapies. *Nat. Rev. Drug Discov.* **19**, 463–479 (2020). 32612263 10.1038/s41573-020-0064-x

[8] Kitada, T., DiAndreth, B., Teague, B. & Weiss, R. Programming gene and engineered-cell therapies with synthetic biology. *Science* **359**, 1067 (2018). 10.1126/science.aad1067 PMC7643872 29439214

[9] Lim, W. A. The emerging era of cell engineering: Harnessing the modularity of cells to program complex biological function. *Science* **378**, 848–852 (2022). 36423287 10.1126/science.add9665

[10] Saka, K., Kawahara, M., Ueda, H. & Nagamune, T. Activation of target signal transducers utilizing chimeric receptors with signaling-molecule binding motifs. *Biotechnol. Bioeng.* **109**, 1528–1537 (2012). 22228507 10.1002/bit.24421

[11] Xie, M. & Fussenegger, M. Designing cell function: assembly of synthetic gene circuits for cell biology applications. *Nat. Rev. Mol. Cell Biol.* **19**, 507–525 (2018). 29858606 10.1038/s41580-018-0024-z

[12] Zhu, L. et al. Engineering a programmed death-ligand 1-targeting monobody via directed evolution for SynNotch-gated cell therapy. *ACS Nano* **18**, 8531–8545 (2024). 38456901 10.1021/acsnano.4c01597 PMC10958600

[13] Ruffo, E. et al. Post-translational covalent assembly of CAR and synNotch receptors for programmable antigen targeting. *Nat. Commun.* **14**, 2463 (2023). 37160880 10.1038/s41467-023-37863-5 PMC10169838

[14] Nakajima, K., Araki, S. & Kawahara, M. Tailoring minimal synthetic receptors to reconstitute signaling properties through multiple tyrosine motifs. *Biochem. Biophys. Res. Commun.* **566**, 148–154 (2021). 34126345 10.1016/j.bbrc.2021.06.014

[15] Fujitani, Y. et al. An alternative pathway for STAT activation that is mediated by the direct interaction between JAK and STAT. *Oncogene* **14**, 751–761 (1997). 9047382 10.1038/sj.onc.1200907

[16] Hintzen, C. et al. Box 2 region of the oncostatin M receptor determines specificity for recruitment of Janus kinases and STAT5 activation. *J. Biol. Chem.* **283**, 19465–19477 (2008). 18430728 10.1074/jbc.M710157200

[17] Ishizuka, S. et al. Designing motif-engineered receptors to elucidate signaling molecules important for proliferation of hematopoietic stem cells. *ACS Synth. Biol.* **7**, 1709–1714 (2018). 29920201 10.1021/acssynbio.8b00163

[18] Kaneko, E., Kawahara, M., Ueda, H. & Nagamune, T. Growth control of genetically modified cells using an antibody/c-Kit chimera. *J. Biosci. Bioeng.* **113**, 641–646 (2012). 22227120 10.1016/j.jbiosc.2011.12.005

[19] Kawahara, M., Hitomi, A. & Nagamune, T. S-Fms signalobody enhances myeloid cell growth and migration. *Biotechnol. J.* **9**, 954–961 (2014). 24376185 10.1002/biot.201300346

[20] Kawahara, M., Ogo, Y., Ueda, H. & Nagamune, T. Improved growth response of antibody/receptor chimera attained by the engineering of transmembrane domain. *Protein Eng. Des. Sel.* **17**, 715–719 (2004). 15548567 10.1093/protein/gzh088

[21] Kawahara, M. et al. Antigen-mediated migration of murine pro-B Ba/F3 cells via an antibody/receptor chimera. *J. Biotechnol.* **133**, 154–161 (2008). 17961783 10.1016/j.jbiotec.2007.09.009

[22] Nakabayashi, H., Aoyama, S., Kawahara, M. & Nagamune, T. Differentiation signalobody: Demonstration of antigen-dependent osteoclast differentiation from a progenitor cell line. *J. Biosci. Bioeng.* **122**, 357–363 (2016). 26979343 10.1016/j.jbiosc.2016.02.010

[23] Nakabayashi, H., Kawahara, M. & Nagamune, T. Cell-surface expression levels are important for fine-tuning the performance of receptor tyrosine kinase-based signalobodies. *Biotechnol. J.* **12**, 1700441 (2017). 10.1002/biot.201700441 28881109

[24] Nakajima, K., Nakabayashi, H. & Kawahara, M. Cell fate-inducing CARs orthogonally control multiple signaling pathways. *Biotechnol. J.* **17**, e2100463 (2022). 35072342 10.1002/biot.202100463

[25] Nakajima, K., Shen, Z., Miura, M., Nakabayashi, H. & Kawahara, M. Sequential control of myeloid cell proliferation and differentiation by cytokine receptor-based chimeric antigen receptors. *PLoS ONE* **17**, e0279409 (2022). 36574389 10.1371/journal.pone.0279409 PMC9794043

[26] Sogo, T. et al. T cell growth control using hapten-specific antibody/interleukin-2 receptor chimera. *Cytokine* **46**, 127–136 (2009). 19223197 10.1016/j.cyto.2008.12.020

[27] Tone, Y., Kawahara, M., Kawaguchi, D., Ueda, H. & Nagamune, T. Death signalobody: inducing conditional cell death in response to a specific antigen. *Hum. Gene Ther. Methods* **24**, 141–150 (2013). 23470213 10.1089/hgtb.2012.147

[28] Ferrao, R. & Lupardus, P. J. The janus kinase (JAK) FERM and SH2 domains: Bringing specificity to JAK-receptor interactions. *Front. Endocrinol. (Lausanne)* **8**, 71 (2017). 28458652 10.3389/fendo.2017.00071 PMC5394478

[29] Seiffert, P. et al. Orchestration of signaling by structural disorder in class 1 cytokine receptors. *Cell Commun. Signal* **18**, 132 (2020). 32831102 10.1186/s12964-020-00626-6 PMC7444064

[30] Tanner, J. W., Chen, W., Young, R. L., Longmore, G. D. & Shaw, A. S. The conserved box 1 motif of cytokine receptors is required for association with JAK kinases. *J. Biol. Chem.* **270**, 6523–6530 (1995). 7896787 10.1074/jbc.270.12.6523

[31] Zhao, W. et al. A conserved IFN-alpha receptor tyrosine motif directs the biological response to type I IFNs. *J. Immunol.* **180**, 5483–5489 (2008). 18390731 10.4049/jimmunol.180.8.5483

[32] Floss, D. M., Moll, J. M. & Scheller, J. IL-12 and IL-23-close relatives with structural homologies but distinct immunological functions. *Cells* **9**, 2184 (2020). 32998371 10.3390/cells9102184 PMC7600943

[33] Ryan, J. J., McReynolds, L. J., Huang, H., Nelms, K. & Paul, W. E. Characterization of a mobile Stat6 activation motif in the human IL-4 receptor. *J. Immunol.* **161**, 1811–1821 (1998). 9712048

[34] Smith, M. J., Hardy, W. R., Murphy, J. M., Jones, N. & Pawson, T. Screening for PTB domain binding partners and ligand specificity using proteome-derived NPXY peptide arrays. *Mol. Cell. Biol.* **26**, 8461–8474 (2006). 16982700 10.1128/MCB.01491-06 PMC1636785

[35] Kongkrongtong, T., Sumigama, Y., Nagamune, T. & Kawahara, M. Reprogramming signal transduction through a designer receptor tyrosine kinase. *Commun. Biol.* **4**, 752 (2021). 34140621 10.1038/s42003-021-02287-8 PMC8211861

[36] Kongkrongtong, T., Zhang, R. & Kawahara, M. Rational design of heterodimeric receptors capable of activating target signaling molecules. *Sci. Rep.* **11**, 16809 (2021). 34413422 10.1038/s41598-021-96396-3 PMC8376883

