# Supplementary material for: Refining minimal engineered receptors for specific activation of on-target signaling molecules
Source: Sci Rep. 2024 Dec 30;14:31671. doi: 10.1038/s41598-024-81259-4 (PMC11685922; doi:10.1038/s41598-024-81259-4)
Supplement: Supplementary file 1 — Supplementary Information 1. [file 41598_2024_81259_MOESM1_ESM.pdf]

Supplementary Information

Refining minimal engineered receptors for specific activation of on-target signaling molecules

Masashi Miura[1], Masahiro Kawahara[1,*]

[1]Laboratory of Cell Vaccine, Microbial Research Center for Health and Medicine (MRCHM), National Institutes of Biomedical Innovation, Health and Nutrition (NIBIOHN), 7-6-8 Saito-Asagi, Ibaraki-shi, Osaka 567-0085, Japan.

[*]Correspondence: Masahiro Kawahara, Laboratory of Cell Vaccine, Microbial Research Center for Health and Medicine (MRCHM), National Institutes of Biomedical Innovation, Health and Nutrition (NIBIOHN), 7-6-8 Saito-Asagi, Ibaraki-shi, Osaka 567-0085, Japan.
E-mail: m-kawahara@nibiohn.go.jp

a) Myristoylated type (Myr)

MGSSKSKPKDPSQRGSGGVQVETISPGDGRTFPKRGQTCVVHYTGMLEDGKKVDSS
RDRNKPFKFMLGKQEVIRGWEEGVAQMSVGQRAKLTISPDYAYGATGHPGIIPPHATL
VFDVELLKLEGSGRWQFPAHYRRLRHALWPSLPDLHRVLGQYLRDTAALSPPKATVS
DTCEEVEPSLLEILPKSSERTPLPLmotif_sequenceIDEQKLISEEDL

b) Cytosolic type (Cyt)

MGVQVETISPGDGRTFPKRGQTCVVHYTGMLEDGKKVDSSRDRNKPFKFMLGKQEV
IRGWEEGVAQMSVGQRAKLTISPDYAYGATGHPGIIPPHATLVFDVELLKLEGSGRWQ
FPAHYRRLRHALWPSLPDLHRVLGQYLRDTAALSPPKATVSDTCEEVEPSLLEILPKSS
ERTPLPLmotif_sequenceIDEQKLISEEDL

c) Transmembrane type (TM)

METDTLLLWVLLLWVPGSTGDYPYDVPDYAGSGGVQVETISPGDGRTFPKRGQTCV
VHYTGMLEDGKKVDSSRDRNKPFKFMLGKQEVIRGWEEGVAQMSVGQRAKLTISPD
YAYGATGHPGIIPPHATLVFDVELLKLEGSGVLLDAPVGLVARLADESGHVVLRWLPP
PETPMTSHIRYEVDVSAGNGAGSVQRVEILEGRTECVLSNLRGRTRYTFAVRARMAEP
SFGGFWSAWSEPVSLLTPSDLDPDIISLVTALHLVLGLSAVLGLLLLRWQFPAHYRRLR
HALWPSLPDLHRVLGQYLRDTAALSPPKATVSDTCEEVEPSLLEILPKSSERTPLPLmoti
f_sequenceIDEQKLISEEDL

STAT3-binding motif

VVHSGYRHQVPS

Y to F mutation of STAT3-binding motif (STAT3F)

VVHSGFRHQVPS

Supplementary Figure 1. The amino acid sequences of MERs examined in main Fig. 1.
Each motif sequence was inserted into the motif_sequence region in the Myr, Cyt, and TM types.
Black: Myristoylation signal sequence. Grey: linker sequence. Light green: $FKBP_{F36V}$. Dark blue: JAK-binding domain of c-mpl. Red: motif sequence. Purple: Myc tag. Brown: leader sequence derived from immunoglobulin κ chain. Orange: HA tag. Yellow: D2 domain of erythropoietin receptor. Light blue: transmembrane domain of c-mpl.

a) Myristoylated type (Myr) with STAT3-binding motif
MGSSKSKPKDPSQRGSGGVQVETISPGDGRTFPKRGQTCVVHYTGMLEDGKKVDSS
RDRNKPFKFMLGKQEVIRGWEEGVAQMSVGQRAKLTISPDYAYGATGHPGIIPPHATL
VFDVELLKLEGSGJAK-binding_domainVVHSGYRHQVPSIDEQKLISEEDL

b) Cytosolic type (Cyt) with STAT3-binding motif
MGVQVETISPGDGRTFPKRGQTCVVHYTGMLEDGKKVDSSRDRNKPFKFMLGKQEV
IRGWEEGVAQMSVGQRAKLTISPDYAYGATGHPGIIPPHATLVFDVELLKLEGSGJAK-
binding_domainVVHSGYRHQVPSIDEQKLISEEDL

intact
RWQFPAHYRRLRHALWPSLPDLHRVLGQYLRDTAALSPPKATVSDTCEEVEPSLLEIL
PKSSERTPLPL

1F
RWQFPAHFRRLRHALWPSLPDLHRVLGQYLRDTAALSPPKATVSDTCEEVEPSLLEILP
KSSERTPLPL

2F
RWQFPAHYRRLRHALWPSLPDLHRVLGQFLRDTAALSPPKATVSDTCEEVEPSLLEILP
KSSERTPLPL

1F, 2F
RWQFPAHFRRLRHALWPSLPDLHRVLGQFLRDTAALSPPKATVSDTCEEVEPSLLEILP
KSSERTPLPL

Supplementary Figure 2. The amino acid sequences of MERs examined in main Fig. 2. Each JAK-binding domain with the Y to F mutation(s) (colored in red) was inserted into the JAK-binding_domain region in Myr / Cyt with the STAT3-binding motif. Color code is the same as Supplementary Fig. 1.

a) Cytosolic type (Cyt) with STAT3-binding motif

MGVQVETISPGDGRTFPKRGQTCVVHYTGMLEDGKKVDSSRDRNKPFKFMLGKQEV
IRGWEEGVAQMSVGQRAKLTISPDYAYGATGHPGIIPPHATLVFDVELLKLEGSGJAK-
binding_domainVVHSGYRHQVPSIDEQKLISEEDL

intact

RWQFPAHFRRLRHALWPSLPDLHRVLGQFLRDTAALSPPKATVSDTCEEVEPSLLEILP
KSSERTPLPL

1L→A

RWQFPAHFRRLRHAAWPSLPDLHRVLGQFLRDTAALSPPKATVSDTCEEVEPSLLEILP
KSSERTPLPL

W→A

RWQFPAHFRRLRHALAPSLPDLHRVLGQFLRDTAALSPPKATVSDTCEEVEPSLLEILP
KSSERTPLPL

1P→A

RWQFPAHFRRLRHALWASLPDLHRVLGQFLRDTAALSPPKATVSDTCEEVEPSLLEILP
KSSERTPLPL

2L→A

RWQFPAHFRRLRHALWPSAPDLHRVLGQFLRDTAALSPPKATVSDTCEEVEPSLLEILP
KSSERTPLPL

2P→A

RWQFPAHFRRLRHALWPSLADLHRVLGQFLRDTAALSPPKATVSDTCEEVEPSLLEILP
KSSERTPLPL

3L→A

RWQFPAHFRRLRHALWPSLPDAHRVLGQFLRDTAALSPPKATVSDTCEEVEPSLLEILP
KSSERTPLPL

Supplementary Figure 3. The amino acid sequences of MERs examined in main Fig. 3. Each JAK-binding domain with the 1F, 2F mutations and one of the Box1 mutations (both colored in red; Box1 filled in yellow) was inserted into the JAK-binding_domain region in Cyt with the STAT3-binding motif. Color code is the same as Supplementary Fig. 1.

a) Cyt (JAK-bd 1F, 2F / Box1 1L→A)
MGVQVETISPGDGRTFPKRGQTCVVHYTGMLEDGKKVDSSRDRNKPFKFMLGKQEV
IRGWEEGVAQMSVGQRAKLTISPDYAYGATGHPGIIPPHATLVFDVELLKLEGSGRWQ
FPAHFRRLRHAAWPSLPDLHRVLGQFLRDTAALSPPKATVSDTCEEVEPSLLEILPKSS
ERTPLPLmotif_sequenceIDEQKLISEEDL

STAT1-binding motif
PTSFGYDKPHVL

STAT2-binding motif
DVDLGDGYIMR

STAT3-binding motif
VVHSGYRHQVPS

STAT4-binding motif
PTHDGYLPSNID

STAT5-binding motif
LMDNAYFCEAD

STAT6-binding motif
PGEAGYKAFSSL

Shc-binding motif
IPVIENPQYFGI

Supplementary Figure 4. The amino acid sequences of MERs examined in main Figs. 4 and 5.

Each motif sequence was inserted into the motif_sequence region in Cyt (JAK-bd 1F, 2F / Box1 1L→A). Color code is the same as Supplementary Fig. 1.

Antibody	Manufacturer	Cat#
rabbit anti-phospho-STAT1(Y701)	Cell Signaling Technology	9171
rabbit anti-STAT1	Cell Signaling Technology	9172
rabbit anti-phospho-STAT2 (Y690)	Thermo Fisher Scientific	PA5-97361
rabbit anti-STAT2	Cell Signaling Technology	72604
rabbit anti-phospho-STAT3 (Y705)	Cell Signaling Technology	9145
rabbit anti-STAT3	Santa Cruz Biotechnology	sc-482
rabbit anti-phospho-STAT4 (Y693)	Cell Signaling Technology	4134
rabbit anti-STAT4	Cell Signaling Technology	2653
rabbit anti-phospho-STAT5 (Y694)	Cell Signaling Technology	9351
rabbit anti-STAT5	Santa Cruz Biotechnology	sc-835
rabbit anti-phospho-STAT6 (Y641)	Cell Signaling Technology	56554
rabbit anti-STAT6	Cell Signaling Technology	5397
rabbit anti-phospho-Shc (Y239/240)	Cell Signaling Technology	2434
rabbit anti-Shc	Santa Cruz Biotechnology	sc-1695
rabbit anti-phospho-Akt (S473)	Cell Signaling Technology	4060
rabbit anti-Akt	Cell Signaling Technology	9272
rabbit anti-phospho-MEK1/2 (S217/221)	Cell Signaling Technology	9154
rabbit anti-MEK1/2	Cell Signaling Technology	8727
rabbit anti-Myc tag	Bethyl Laboratories	A190-105A
rabbit anti-GAPDH	Cell Signaling Technology	5174
HRP-conjugated goat anti-rabbit IgG	Thermo Fisher Scientific	G-21234

Supplementary Figure 5 The antibodies used in Western blotting.

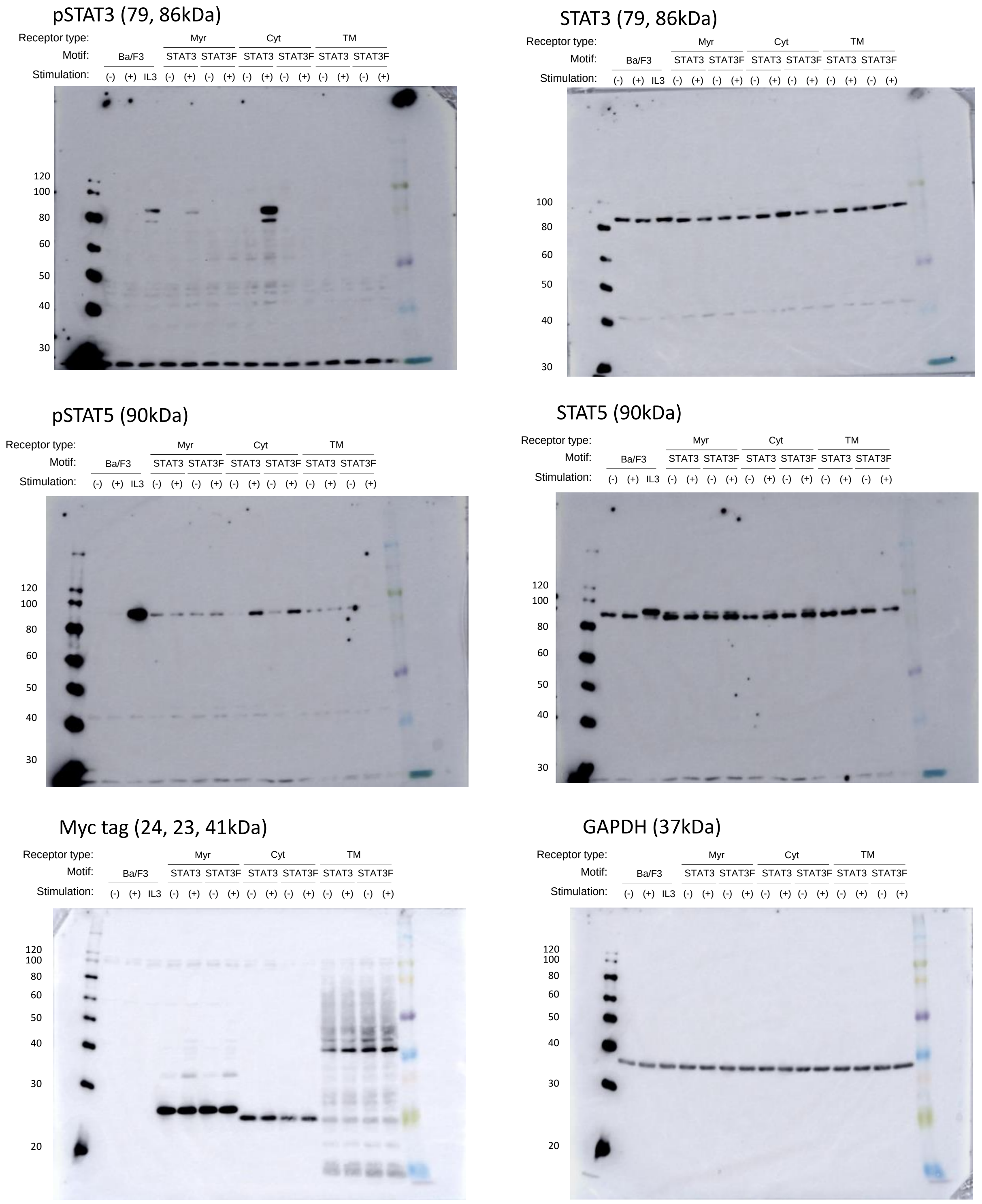

Supplementary Figure 6. Uncropped blot images for Fig. 1b.

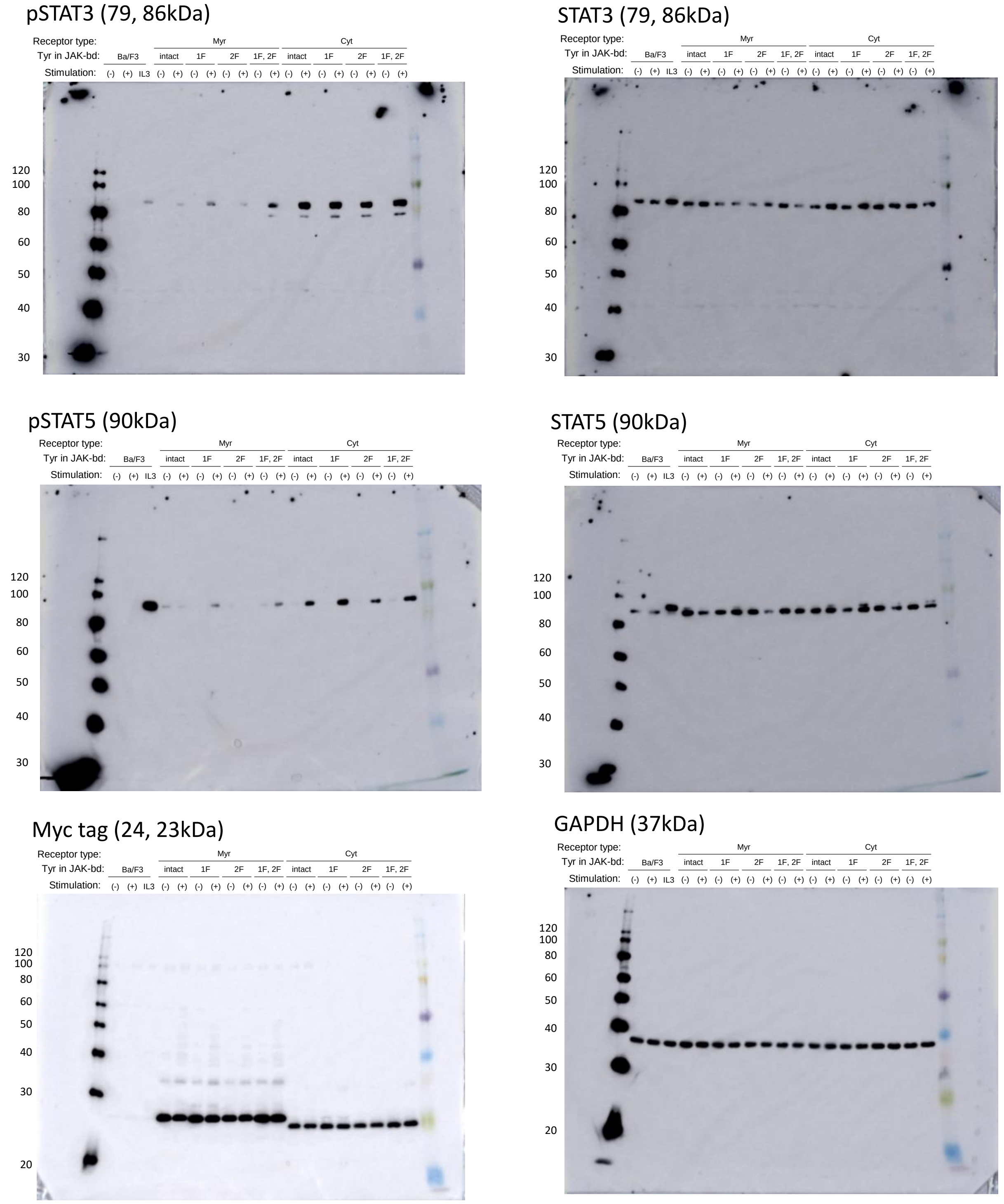

Supplementary Figure 7. Uncropped blot images for Fig. 2b.

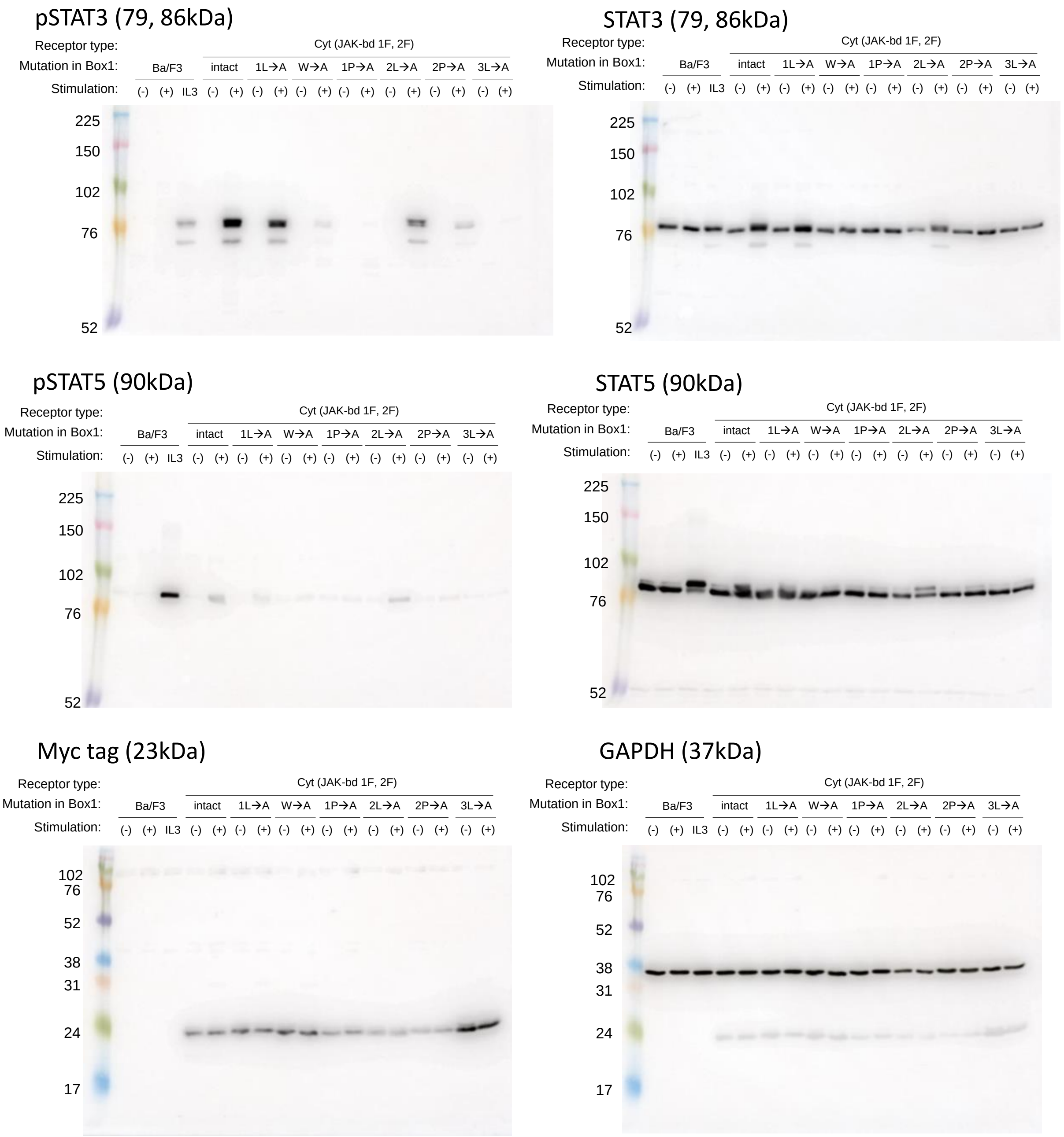

Supplementary Figure 8. Uncropped blot images for Fig. 3b.

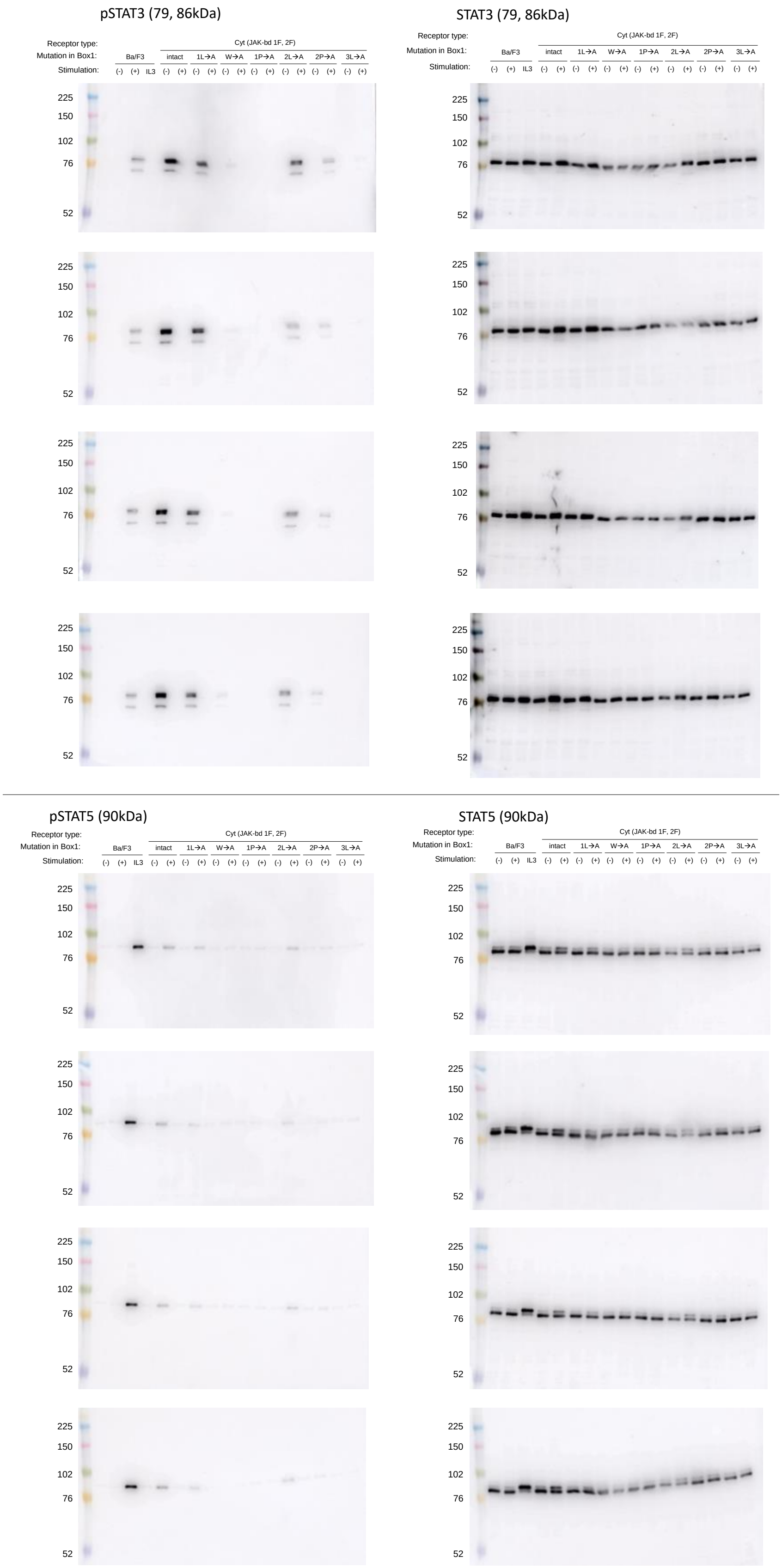

Supplementary Figure 9. Uncropped blot images for the other four replicate blots of pSTAT3/whole STAT3 and pSTAT5/whole STAT5 with regard to Fig. 3c.

pSTAT1 (84, 91kDa)

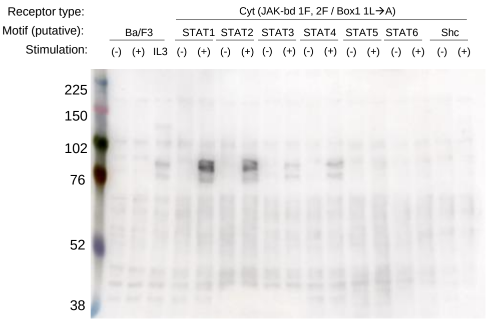

STAT1 (84, 91kDa)

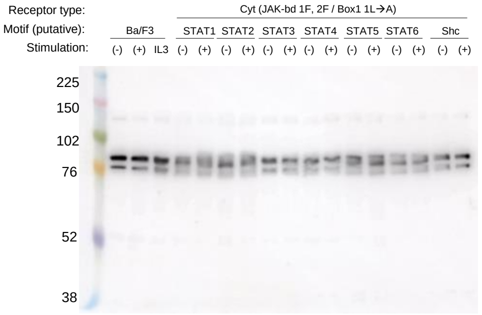

pSTAT2 (130kDa)

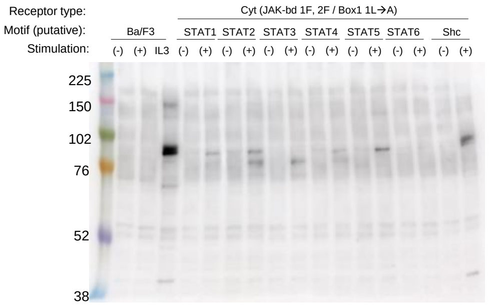

STAT2 (130kDa)

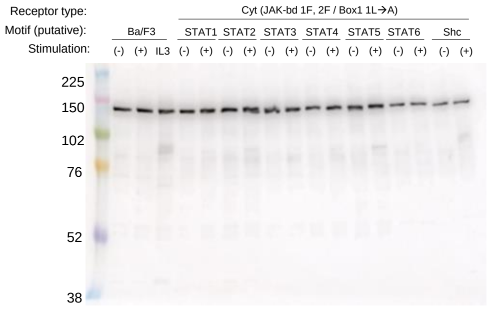

pSTAT3 (79, 86kDa)

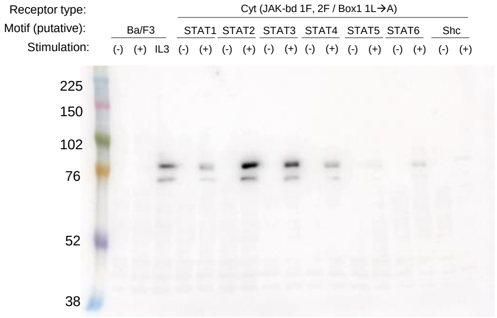

STAT3 (79, 86kDa)

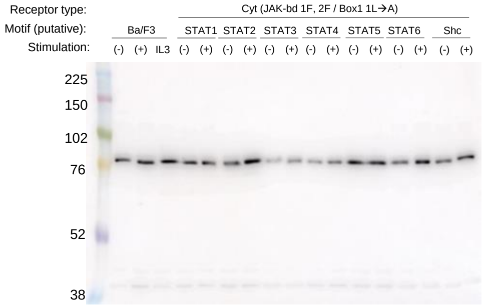

pSTAT4 (81kDa)

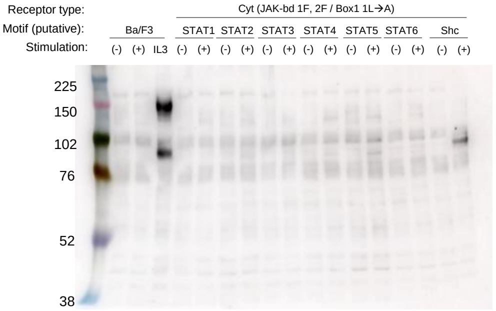

STAT4 (81kDa)

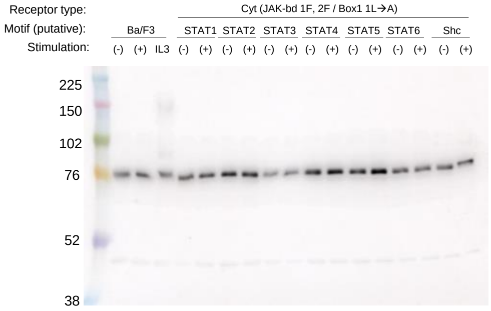

pSTAT5 (90kDa)

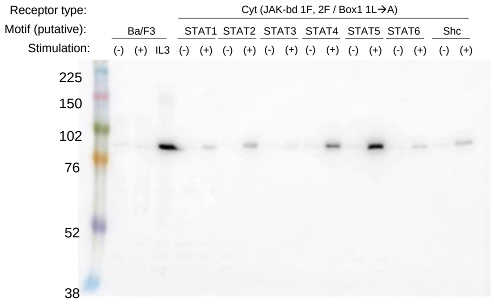

STAT5 (90kDa)

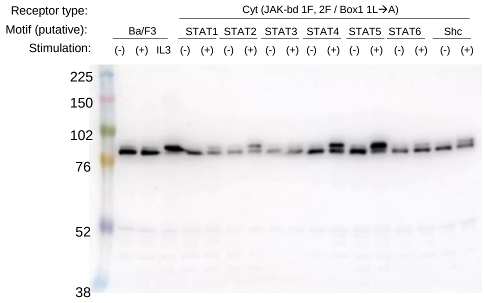

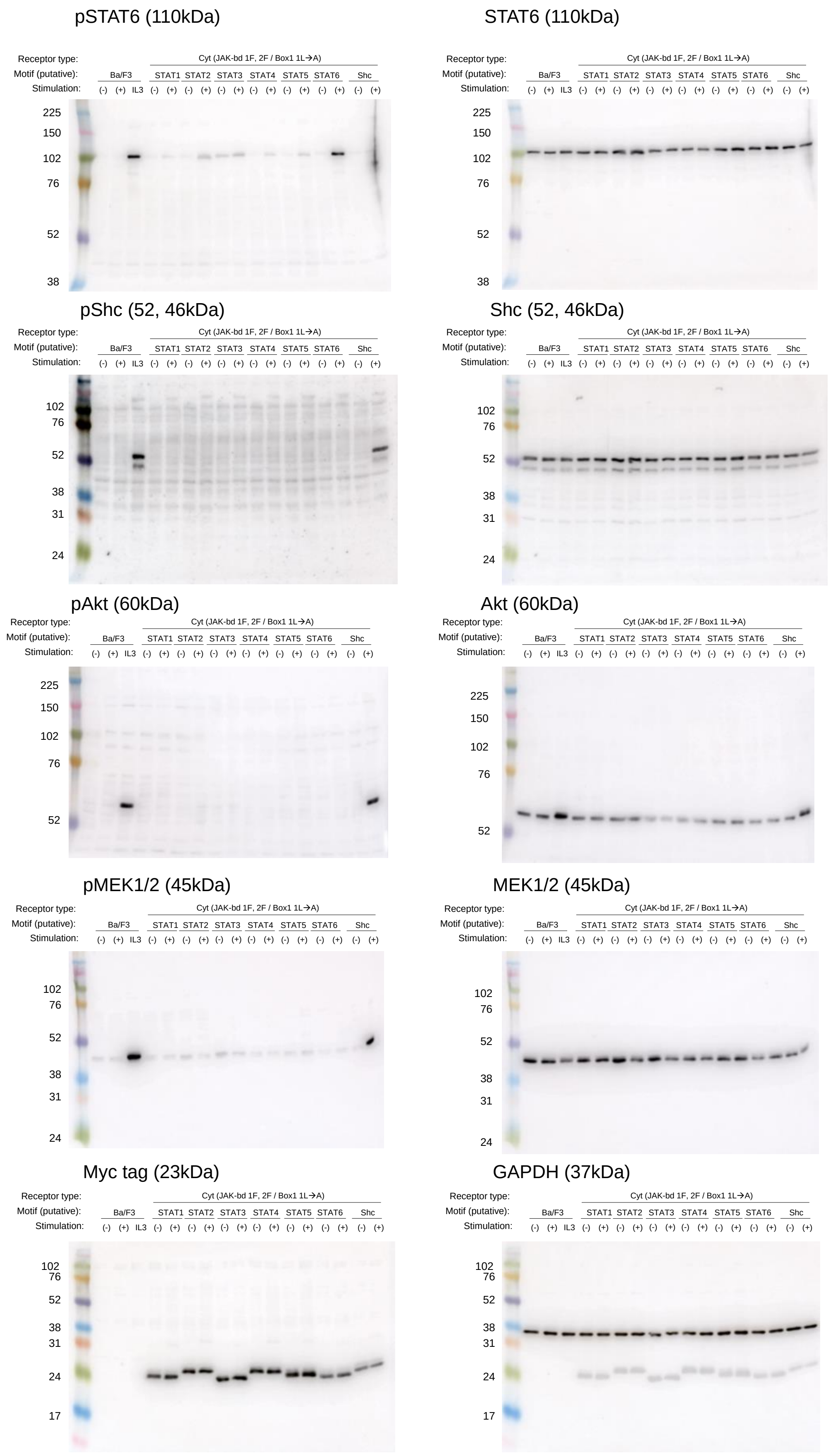

Supplementary Figure 10. Uncropped blot images for Fig. 4.

	Motif(putative)						
	STAT1	STAT2	STAT3	STAT4	STAT5	STAT6	Shc
pSTAT1	+++	+++	+	+			
pSTAT2							
pSTAT3	+	+++	+++	+		+	
pSTAT4							
pSTAT5	+	+		++	+++	+	+
pSTAT6	+	+	+	+	+	+++	
pShc							+++
pAkt							+++
pMEK							+++

Supplementary Figure 11. Activation patterns of signaling molecules induced by the MERs tested in Fig. 4.

Activation levels are represented as the number of '+'.

Description of Supplementary Movies

Supplementary Movie 1
Time-lapse observation of parental Ba/F3 cells without the ligand (-).

Supplementary Movie 2
Time-lapse observation of parental Ba/F3 cells with 50 nM AP20187 (+).

Supplementary Movie 3
Time-lapse observation of cells expressing the MER incorporating the STAT1-binding motif without the ligand (-).

Supplementary Movie 4
Time-lapse observation of cells expressing the MER incorporating the STAT1-binding motif with 50 nM AP20187 (+).

Supplementary Movie 5
Time-lapse observation of cells expressing the MER incorporating the STAT2-binding motif without the ligand (-).

Supplementary Movie 6
Time-lapse observation of cells expressing the MER incorporating the STAT2-binding motif with 50 nM AP20187 (+).

Supplementary Movie 7
Time-lapse observation of cells expressing the MER incorporating the STAT3-binding motif without the ligand (-).

Supplementary Movie 8
Time-lapse observation of cells expressing the MER incorporating the STAT3-binding motif with 50 nM AP20187 (+).

Supplementary Movie 9
Time-lapse observation of cells expressing the MER incorporating the STAT4-binding motif without the ligand (-).

Supplementary Movie 10
Time-lapse observation of cells expressing the MER incorporating the STAT4-binding motif with 50 nM AP20187 (+).

Supplementary Movie 11
Time-lapse observation of cells expressing the MER incorporating the STAT5-binding motif without the ligand (-).

Supplementary Movie 12
Time-lapse observation of cells expressing the MER incorporating the STAT5-binding motif with 50 nM AP20187 (+).

Supplementary Movie 13
Time-lapse observation of cells expressing the MER incorporating the STAT6-binding motif without the ligand (-).

Supplementary Movie 14
Time-lapse observation of cells expressing the MER incorporating the STAT6-binding motif with 50 nM AP20187 (+).

Supplementary Movie 15
Time-lapse observation of cells expressing the MER incorporating the Shc-binding motif without the ligand (-).

Supplementary Movie 16
Time-lapse observation of cells expressing the MER incorporating the Shc-binding motif with 50 nM AP20187 (+).
